# Characterization of the φCTX-like *Pseudomonas aeruginosa* phage Dobby isolated from the kidney stone microbiota

**DOI:** 10.1099/acmi.0.000002

**Published:** 2019-03-20

**Authors:** Genevieve Johnson, Alan J. Wolfe, Catherine Putonti

**Affiliations:** 1 Bioinformatics Program, Loyola University Chicago, Chicago, IL, USA; 2 Department of Microbiology and Immunology, Loyola University Chicago, Maywood, IL, USA; 3 Department of Biology, Loyola University Chicago, Chicago, IL, USA; 4 Department of Computer Science, Loyola University Chicago, Chicago, IL, USA

**Keywords:** *Pseudomonas *phage φCTX, kidney stones, *Pseudomonas aeruginosa*

## Abstract

Bacteriophages (phages) are vital members of the human microbiota. They are abundant even within low biomass niches of the human body, including the lower urinary tract. While several prior studies have cultured bacteria from kidney stones, this is the first study to explore phages within the kidney stone microbiota. Here we report Dobby, a temperate phage isolated from a strain of *Pseudomonas aeruginosa* cultured from a kidney stone. Dobby is capable of lysing clinical *P. aeruginosa* strains within our collection from the urinary tract. Sequencing was performed producing a 37 152 bp genome that closely resembles the temperate *P. aeruginosa* phage φCTX, a member of the P2 phage group. Dobby does not, however, encode for the cytotoxin CTX. Dobby’s genome was queried against publicly available bacterial sequences identifying 44 other φCTX-like prophages. These prophages are integrated within the genomes of *P. aeruginosa* strains from a variety of environments, including strains isolated from urine samples and other niches of the human body. Phylogenetic analysis suggests that the temperate φCTX phage species is widespread. With the isolation of Dobby, we now have evidence that phages are members of the kidney stone microbiota. Further investigation, however, is needed to determine their abundance and diversity within these communities.

## Impact Statement

Here we report Dobby, a temperate phage isolated from a strain of *Pseudomonas aeruginosa* cultured from a kidney stone. This is the first phage isolated from the kidney stone microbiota, providing evidence that this low biomass community contains bacteria as well as phages. While the complete genome of Dobby is most similar to the *Pseudomonas* phage φCTX, it does not encode for a cytotoxin. Other φCTX-like phage sequences, exhibiting sequence homology to Dobby, were identified in publicly available *Pseudomonas* genomes from clinical isolates, livestock, soil and water, revealing the prevalence of this phage species. Dobby is, however, unique from φCTX-like phages isolated from urine thus prompting subsequent studies of phage populations within the urinary tract.

## Data Summary

Raw sequencing reads have been deposited as part of BioProject PRJNA494532 in NCBI’s SRA database (SRR7959260) and the complete genome sequence has been deposited in GenBank (MK034952). The phage strain Dobby is available upon request from the authors.

Bacteria can cause magnesium–ammonium–phosphate (struvite) kidney stones [1]. However, struvite stones, caused by urinary tract infections, account for only 4% of urinary stones [2]. Much more frequently, kidney stones are formed by calcium, either oxalate (CaOx) or calcium phosphate (CaPhos) [3]. Evidence suggests that bacteria also may contribute to calcium kidney stones [4–7], as several studies have succeeded in isolating viable bacteria [4–10]. *Escherichia coli* and *Pseudomonas* spp. are the most common bacteria cultured from kidney stones [2]. In Barr-Beare *et al.* [7], viable bacteria were cultured from two stones, although bacterial DNA was detected via 16S rRNA gene sequencing in all five stones examined. Thus, it is likely that the microbiota of kidney stones, like that of bladder urine, is a low biomass community. As recent evidence has found bacteriophages (phages) within the urinary microbiota [11–14], it is likely that phages are also members of the kidney stone microbiota. Here, we present the first phage isolated from the kidney stone microbiota.

*Pseudomonas* phage Dobby was isolated from *P. aeruginosa* strain UMB2738 cultured from a CaOx kidney stone (from [7]). The bacterial strain was isolated using the Expanded Quantitative Urinary Culture (EQUC) protocol [15] and stored at −80 °C. We streaked a 1.7% LB agar plate with a loop from this freezer stock. A single colony of *P. aeruginosa* UMB2738 was selected from a plate and grown in LB overnight at 37 °C, shaking (with 5 mm sterile glass beads to minimize biofilm formation). The culture was then plated (3 ml 0.7% LB soft agar +1 ml bacteria culture, poured on a 1.7% LB agar plate) and grown overnight at 37 °C. Plaques were found on the bacterial lawn and harvested, suspended in LB, and filtered through a 0.2 µm cellulose acetate syringe filter. The phage lysate was then spotted onto *P. aeruginosa* lawns of our collection of 21 other *P. aeruginosa* strains isolated by EQUC from the urinary tract and the laboratory strain *P. aeruginosa* ATCC 15692. Lysis was observed in 19 of the 22 clinical strains tested, but not for the laboratory strain.

The phage lysate was regrown in an overnight culture of one of our clinical strains (UMB1204) in which clear lysis was observed during plating. The inoculated culture was grown overnight at 37 °C, shaking (with 5 mm sterile glass beads), and then spun down. Lysate was removed and treated with OPTIZYME DNase I (Fisher BioReagents) prior to DNA extraction using the Quick-DNA kit (Zymo). The DNA library was prepared using the Nextera XT WGS kit and sequenced on the Illumina MiSeq platform (v2 Reagent Kit, 2×250), producing 1 596 454 read pairs. The raw reads were trimmed using sickle (https://github.com/najoshi/sickle) and then assembled using SPAdes v 3.11.1 with the meta flag [16]. The assembled contigs were queried locally via blastn against RefSeq *P. aeruginosa* genome sequences. This facilitated the identification of the single contig representative of the phage’s genome. Other contigs assembled represented sequenced host DNA, exhibiting sequence homology to *P. aeruginosa* genome sequences. The phage genome had a sequencing depth (coverage=864x) over tenfold greater than other assembled contigs. The genome sequence was annotated using RAST [17].

Dobby’s complete genome is 37 152 bp in length with a GC content of 62.2% and includes 49 predicted coding regions. The genome sequence was queried online using blastn against NCBI’s viral sequences within the nr/nt database, identifying bacteriophage φCTX (GenBank accession AB008550 [18]) as its closest relative (nucleotide sequence identity=94%; query coverage=75%). This temperate phage encodes for the cytotoxin gene *ctx*. First isolated from *P. aeruginosa* strain PA158, φCTX was found capable of converting non-CTX producing *P. aeruginosa* strains into CTX producers [19] and *P. aeruginosa* strains harbouring this phage are more virulent than those without [20]. Prior research determined the host receptor for φCTX; O-serotype *P. aeruginosa* strains are sensitive to the phage [21]. φCTX is in the P2 phage group; its virion assembly genes are syntenic with those of the *E. coli *-infecting phage P2 [22] and Dobby. However, Dobby does not encode for *ctx*.

**Fig. 1.**
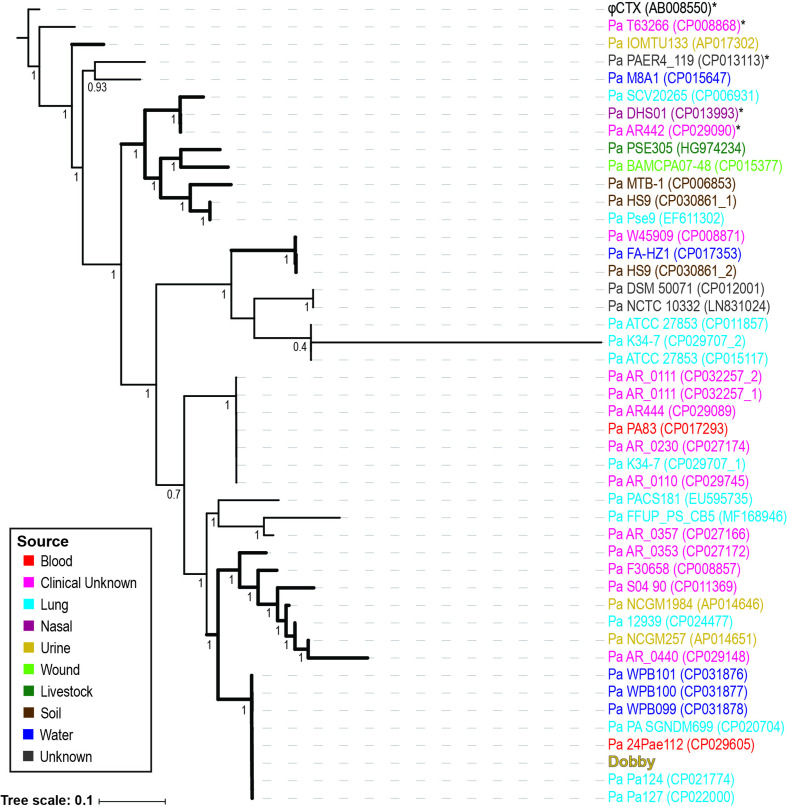
Genome sequence similarity amongst φCTX-like phages, coloured according to the location from which the strain was isolated. ‘Pa’ indicates prophage sequence within a *P. aeruginosa* genome. Sequences with * include the cytotoxin gene. Branches emphasized with a heavier line indicate strains encoding for the same integrase as Dobby. Local support values are indicated on the tree.

Numerous hits were detected between Dobby and *P. aeruginosa* genomes (query coverage >60%) in the nr/nt database. For each of these *P. aeruginosa* genomes, the intact prophage sequences were retrieved using PHASTER [23]. In total, 44 prophage sequences were identified; three genomes had two intact φCTX-like prophage sequences. The prophage sequences, the φCTX genome and Dobby’s genome were first compared using mauve [24] to identify syntenic blocks. The sequences were then aligned using Clustal Omega [25], organized into a phylogenetic tree using FastTree [26], and visualized using iTOL [27] (Fig. 1).

Dobby includes nine predicted coding regions that are not found within the φCTX genome: seven hypothetical proteins, a phosphoadenosine phosphosulfate reductase, and an integrase. These nine predicted coding regions, however, are present in many of the prophage sequences examined in our phylogenetic analysis. While φCTX is classified as a P2-like phage, its integrase does not show significant sequence similarity to P2; rather it most closely resembles the Rac prophage of *E. coli* K-12 [18]. Further, Dobby encodes for an integrase distinct from φCTX and P2. Dobby’s integrase, however, is also present within several of the prophages identified here (Fig. 1). The nucleotide sequence for Dobby’s integrase was queried against the nr/nt database revealing sequence homology to other *Pseudomonas* strains. Further investigation of these hits found prophage sequences encoding for several genes found within φCTX. But with a query coverage <30%, these hits represent a distinctly different viral lineage.

Here, we have identified φCTX-like phage sequences isolated from a variety of sources, including environmental samples and various sites of the human body (Fig. 1). This suggests that φCTX-like phages are likely to persist wherever *P. aeruginosa* is found. Nevertheless, most of the prophage sequences do not encode for *ctx*. While Dobby was isolated from a strain from the urinary tract, it does not resemble φCTX-like phages from other urinary samples (Fig. 1). Nucleotide sequence similarity to these other urinary φCTX-like phages is 59, 73 and 88 % to *P. aeruginosa* IOMTU133 (AP017302), NCGM257 (AP014651) and NCGM1984 (AP014646), respectively. Thus, φCTX-like phages within the urinary microbiota also vary. As shown in Fig. 1, Dobby’s genome sequence is most similar to prophages within the genomes of three *P. aeruginosa* strains isolated from hospital wastewater (shown in dark blue), three strains from the lung (sputum/bronchial washing) (shown in light blue) and one strain from blood (shown in red). Dobby’s genome, however, is significantly shorter than the four prophage sequences from the other clinical samples (40.6 kbp), which encode for the O antigen gene cluster *rfbA, rfbB* and *rfbD* at the 3′ end. This same gene cluster is adjacent to the PHASTER-predicted prophages within the genomes of the three strains from hospital wastewater. Thus, in some cases PHASTER included these genes as part of the prophage (the clinical samples), while in others (the wastewater samples) it did not. By isolating Dobby in the lytic phase, we can definitively show that these genes are in fact not encoded by the phage. Rather it is likely that the phage was integrated into the *Pseudomonas* genome with the *rfb* genes, which are believed to have been acquired by horizontal gene transfer [28]. These genes have previously been associated with *Pseudomonas* resistance to phage [29].

The sequencing of *Pseudomonas* phage Dobby prompted our larger examination of *P. aeruginosa* φCTX-like prophage sequences, finding similar phages in a variety of environments. This suggests that like pseudomonads, this phage is widespread. Furthermore, the majority (>90%) of the prophage sequences identified do not encode for the cytotoxin and those that do are not monophyletic. The isolation and sequencing of Dobby and our phylogenetic analysis provides insight into the diversity of *Pseudomonas* P2 viruses. More importantly, the temperate phage Dobby provides evidence that kidney stone microbiota can also include phages. Subsequent studies are needed to assess the prevalence of phages within stones and their lytic efficiency.
